# Congenital prosopagnosia: A case report

**DOI:** 10.1590/S1980-57642011DN05010010

**Published:** 2011

**Authors:** Rodrigo Rizek Schultz, Paulo Henrique Ferreira Bertolucci

**Affiliations:** 1 Head, Behavior Neurology Section, University of Santo Amaro, São Paulo SP, Brazil; Behavior Neurology Section, Department of Neurology and Neurosurgery, Escola Paulista de Medicina, Federal University of São Paulo, São Paulo SP, Brazil.; 2 Head, Behavior Neurology Section, Department of Neurology and Neurosurgery, Escola Paulista de Medicina, Federal University of São Paulo, São Paulo SP, Brazil.

**Keywords:** prosopagnosia, congenital, agnosia, face recognition

## Abstract

Prosopagnosia is a visual agnosia characterized by an inability to recognize previously known human faces and to learn new faces. The aim of this study was to present a forty-six year-old woman with congenital prosopagnosia, and to discuss the neural bases of perception and recognition of faces. The patients had a lifetime impairment in recognizing faces of family members, close friends, and even her own face in photos. She also had impairment in recognizing animals such as discriminating between cats and dogs. The patient’s basic visual skills showed impairment in identifying and recognizing the animal form perception on the coding subtest of the WAIS-R, recognizing overlapping pictures (Luria), and in identifying silhouettes depicting animals and objects (VOSP). Unconventional tests using pictures evidenced impairment in her capacity to identify famous faces, facial emotions and animals. Her face perception abilities were preserved, but recognition could not take place. Therefore, it appears that the agnosia in this case best fits the group of categories termed “associative”.

## Abstract (pt)

Prosopagnosia é uma agnosia visual caracterizada por uma incapacidade de reconhecer faces humanas vistas anteriormente e aprender outras. O objetivo é apresentar uma mulher de 46 anos com prosopagnosia congênita e discutir as bases neurais da percepção e do reconhecimento de faces. Ela nos procurou referindo apresentar desde a infância problemas no reconhecimento de faces de membros da família, amigos próximos e mesmo para sua própria imagem numa fotografia. Também diz apresentar prejuízo no reconhecimento de animais, como discriminar cães de gatos. Apresentou dificuldades em identificar e reconhecer animais desenhados; reconhecer figuras sobrepostas (Luria), incorrendo em paragnosias visuais e identificar silhuetas de animais (VOSP). Em testes não convencionais, usando figuras, evidenciou diminuição da capacidade em identificar faces famosas, expressões faciais e animais, mas não em estimar o sexo e a idade das pessoas. Concluindo, suas habilidades perceptuais para face estão preservadas, mas há um déficit de reconhecimento. Tudo indica que sua agnosia pertence ao grupo das associativas.

Visual agnosia is an impairment in recognizing visual stimuli that cannot be explained by sensory loss. Prosopagnosia is a form of visual agnosia characterized by an inability to recognize previously known human faces and to learn new faces.^1^ Congenital prosopagnosia (CP) denotes a deficit in face processing apparent from early childhood in the absence of any underlying neurological basis, in the presence of intact sensory and intellectual function.^2^

A classical distinction made between different forms of prosopagnosia is whether the deficit is “apperceptive” or “associative” in nature. This dichotomy attributes the former condition to a deficit in deriving a sufficiently intact perception, whereas in the latter type, the root of the impairment is at the level of recognition or assignment of meaning.^3,4^

CP contrasts with the more general term “developmental prosopagnosia”, which includes not only individuals with CP but also those who have sustained brain damage either before birth or in early childhood.^2^ Notably, in recent years a growing number of CP cases have been reported, but whether this is due to increasing prevalence or increased recognition of the disorder, is not known. There is growing evidence that a familial factor is involved in many cases of CP and thus, formal testing establishing face processing impairments in additional family members could further assist in the differential diagnosis of CP as opposed to acquired prosopagnosia (AP).^5,6^

Seven family pedigrees with 38 cases in two to four generations of suspected hereditary prosopagnosia have been detected using a screening questionnaire. Men and women are impaired, and the anomaly is regularly transmitted from generation to generation in all pedigrees studied. Segregation is best explained by a simple autosomal dominant mode of inheritance, suggesting that loss of human face recognition can occur by the mutation of a single gene.^7,8^

## Objective

To present a forty-six year-old woman with congenital prosopagnosia and to discuss the neural bases of perception and recognition of faces.

## Case report

The case involves C.M., a forty-six year-old forced right-handed woman. She is an executive and speaks fluent Portuguese and Greek. At the age of forty-five she was referred for neurological evaluation with complaints of facial recognition problems since childhood. She had a lifetime history of impairment in recognizing faces of family members, close friends, and even her own face in photos. The person’s voice easily reveals the identity of unrecognized faces, as do a variety of clues such as clothes, attitude and body build. She also had impairment in recognizing animals, such as discriminating between cats and dogs. C.M. was submitted to a series of neuropsychological and perceptual tests. A complete medical and neurological examination was performed, yet revealed no pathology. To assess other disturbances frequently associated with prosopagnosia such as quadrantonopsia, achromatopsia and topographic agnosia, the subject performed basic tests and answered a few questions, revealing no problems. Finally, she was normal on brain SPECT and brain MRI exams.

### Tests administered

The investigation of C.M.’s cognitive functioning began with application of the WAIS-R.^9^ She had a superior intellectual range in verbal skills and a low average in non-verbal skills. She had intact basic attentional tests for verbal stimuli and a slow performance for visual stimuli. Her basic visual skills showed impairment in identifying and recognizing the animal form perception on the coding subtest of the WAIS-R, in recognizing overlapping pictures (Luria), and in identifying silhouettes depicting animals and objects (VOSP).^10,11^ Unconventional tests using pictures evidenced impairment in her capacity to identify famous faces, facial emotions and animals, but not to identify gender and estimate age. She had a normal memory and no disturbance in constructional tasks.

## Discussion

Prosopagnosia is invariably accompanied by other visual impairments, so it is difficult to determine the extent to which a prosopagnosic’s deficit is limited to facial processing.^12^ The extent to which CP is specific to faces remains the subject of an ongoing controversy in the field of visual cognitive neuroscience. Neuropsychological case studies have demonstrated a double dissociation between the recognition of faces and objects, suggesting independence or segregation between these processes and indicating the existence of a neural system specialized, if not dedicated, to faces. An alternative view, however, also supported by numerous neuropsychological and imaging investigations, is that there is a single, general-purpose visual process that subserves both objects and faces. The controversy between a domain-specific organization of faces versus a more generic system has not been resolved.^2,13^ An interesting view is that when one is asked to name an object or animal (e.g. a chair or a dog), one is not being asked the particular type of object in question. In this sense if one correctly names a stimulus as “a human face” this could be considered at the same level as naming a chair or a dog. The point in prosopagnosia is the requirement to evoke the specific context of the stimulus (who is this person?). As a general rule, human faces have several patterns in common and as such might be considered “ambiguous”.^14^ In this case, associative prosopagnosics like CM, can easily identify a stimulus as a human face, but are unable to identify, within the general group, who that particular person is. There are other groups that are ambiguous, like animals or personal objects. If this is true, an associative prosopagnosic would also have difficulty with these categories. It is interesting that CM, upon reporting her cat running away, remembered that she would call any cat, because she did not know whether it was her cat or not. This issue could be clarified by testing prosopagnosics not only on recognition of faces, but also on ambiguous objects.

Accurate discrimination of familiar faces from unfamiliar ones is an essential biological and social skill that comprises a number of distinct cognitive operations.^15^

Cognitive models of normal visual information processing are increasingly used as frameworks for explaining neuropsychological impairment.^4,16^ Within the framework of the Bruce and Young (1986) model of face recognition, the locus of impairment in “apperceptive” prosopagnosia is at a relatively early stage of face processing where abstract structural descriptions of the encountered face stimuli are generated by the visual system. By contrast, in “associative” prosopagnosia, face perception abilities are preserved, but recognition cannot take place because face recognition units (FRU) are destroyed, or cannot be accessed. Unlike patients with apperceptive prosopagnosia, patients with “associative” prosopagnosia may perform well on face processing tasks that do not require the recognition of facial identity.^15^

Achromatopsia and topographic agnosia are common disturbances in apperceptive prosopagnosia, but these problems were not detected in our case. In this patient, face perception abilities were preserved, but recognition could not take place. Therefore, it seems that her agnosia best fits the group of categories termed “associative”.^17^

Although CP mirrors the characteristics of AP which can be as severely debilitating, the neural basis of AP is well established, whereas the neural origin of CP remains elusive. AP typically occurs in individuals following a lesion such as a bilateral or unilateral right hemisphere stroke in the inferior occipitotemporal cortex and classically implicates the anterior temporal lobe and the fusiform and lingual gyri. In contrast, CP occurs in the absence of any obvious discernible lesion on conventional neuroimaging, or any other neurological concomitant.^18^

Some authors have explored the neurobiological substrate of CP. Given that face processing is mediated by a widely distributed network, comprising “core” regions in the ventral occipitotemporal cortex and “extended” regions in anterior temporal and frontal cortices, it is conceivable that CP might be attributable to abnormal functioning of the core regions. Thus, a plausible alternative hypothesis is that CP stems from a disruption in the structural connectivity between the nodes of the distributed face-processing network. This hypothesis is also consistent with the fact that there is a reduction in the volume of the anterior fusiform gyrus in CP, but whether this reduction is attributable to decreases in gray and/or white matter is not yet known. Using diffusion tensor imaging and tractography, some authors have found that a disruption in structural connectivity in ventral occipitotemporal cortex may be the neurobiological basis for the lifelong impairment in face recognition. These findings suggest that white-matter fibers in ventral occipitotemporal cortex support the integrated function of a distributed cortical network that subserves normal face processing.^19^

Other authors have used voxel-based morphometry to investigate whether developmental prosopagnosics show subtle neuroanatomical differences from controls. An analysis based on segmentation of T1-weighted images from 17 developmental prosopagnosics and 18 matched controls, revealed that the former had reduced grey matter volume in the right anterior inferior temporal lobe and in the superior temporal sulcus/middle temporal gyrus bilaterally. In addition, a voxel-based morphometry analysis, based on the segmentation of magnetization transfer parameter maps, showed that developmental prosopagnosics also had reduced grey matter volume in the right middle fusiform gyrus and the inferior temporal gyrus. Their results demonstrate that developmental prosopagnosics have reduced grey matter volume in several regions known to respond selectively to faces, providing new evidence that integrity of these areas relates to face recognition ability.^20^

To investigate the neural basis of CP, some authors have conducted detailed morphometric and volumetric analyses of the occipitotemporal (OT) cortex in a group of CP individuals and matched control subjects using high-spatial resolution magnetic resonance imaging. Although there were no significant group differences in the depth or deviation from the midline of the OT or collateral sulci, the CP individuals evidenced a larger anterior and posterior middle temporal gyrus and a significantly smaller anterior fusiform (aF) gyrus. Interestingly, this volumetric reduction in the aF gyrus is correlated with the behavioral decrement in face recognition. These findings implicate a specific cortical structure as the neural basis of CP and, in light of the familial history of CP, target the aF gyrus as a potential site for further, focused genetic investigation.^9^

That CP exists at all, coupled with the fact that the deficit in these individuals is not resolved over the course of their lifetime, suggests there is a limit on the plasticity of the human ventral visual system. This is in marked contrast with the rather widespread plasticity reported in other sensory domains as well as in higher-cognitive skills such as language. We should also note that CP might not be as rare as previously thought and, like other developmental disorders, questions concerning intervention and training will become more pressing.^2^
